# Patent Foramen Ovale Percutaneous Closure: Evolution and Ongoing Challenges

**DOI:** 10.3390/jcm13010054

**Published:** 2023-12-21

**Authors:** Perrine Devos, Paul Guedeney, Gilles Montalescot

**Affiliations:** 1 ACTION Study Group, INSERM UMRS_1166 Institut de Cardiologie (AP-HP), Sorbonne Université, 75005 Paris, France; perrine.devos@aphp.fr (P.D.); paul.guedeney@aphp.fr (P.G.); 2 Institut de Cardiologie, Centre Hospitalier Universitaire, Pitié-Salpêtrière, 47 Boulevard de l’Hôpital, 75013 Paris, France

**Keywords:** patent foramen ovale, PFO, cryptogenic stroke, PFO closure, migraine, residual shunt, decompression sickness, orthodeoxia syndrome

## Abstract

Patent foramen ovale (PFO) concerns nearly a quarter of the general population and incidence may reach up to 50% in patients with cryptogenic stroke. Recent randomized clinical trials confirmed that percutaneous closure of PFO-related stroke reduces the risk of embolic event recurrence. PFO also comes into play in other pathogenic conditions, such as migraine, decompression sickness or platypnea–orthodeoxia syndrome, where the heterogeneity of patients is high and evidence for closure is less well-documented. In this review, we describe the current indications for PFO percutaneous closure and the remaining challenges, and try to provide future directions regarding the technique and its indications.

## 1. Introduction

Patent foramen ovale (PFO) is implicated in the pathogenesis of several medical conditions. PFO closure has been established as the cornerstone therapy for patients with cryptogenic stroke and concomitant PFO. In other conditions, such as migraines, decompression sickness or platypnea–orthodeoxia syndrome, evidence for closure is weak. In this review, we summarize the existing evidence to help physicians in the management of these situations. We also underscore the potential complications and remaining challenges of PFO closure.

## 2. Historical Context

Essential to fetal life, foramen ovale is a normal heart structure that aims to direct blood flow from the right to the left atrium, avoiding the pulmonary circulation. Discovered by Galien around the IInd century, its role in fetal life was described for the first time by Eustachi in 1571. PFO can be present in about 25% of the general population [1] and can be responsible for paradoxical embolism—which may suppose that a thrombus from the venous system enters the arterial system via the defect in atrial septum. Mutations of some genes were found to be associated with atrial septal defects in humans (i.e., NKX2-5, GATA4, TBX5, MYH6 or ACTC genes), but, to date, there is no variant associated with PFO [2,3]. In 1988, Lechat et al. were the first to demonstrate a higher prevalence of PFO in patients with cryptogenic stroke; that rate can reach up to 40–50% [4,5,6]. The concept of cryptogenic stroke was developed to characterize patients without risk factors or cause of stroke and included criteria such as lesion size and localization, which resulted in a subgroup called “Embolic Stroke of Undetermined Source” (ESUS) [7].

Nevertheless, the pathophysiologic concept underlying paradoxical embolism is not yet totally elucidated. Indeed, DVT (deep vein thrombosis) can be a potential source for an embolus, but the PFO itself may also represent a prothrombotic structure. In fact, presence of an atrial septum aneurism (ASA) seems to increase the risk of first stroke, with an OR 3.38 in a meta-analysis [8]. As the risk of recurrent stroke in patients with PFO and ASA is higher than it is in patients without PFO or ASA (15.2% [1.8–28.6] vs. 4.2% [1.8–6.6] at 4 years) [9], closing PFO appeared as an attractive strategy to lower this risk. Catheter-based closure of PFO was introduced in 1992 [10], and observational data showed that closure of PFO in patients with a history of stroke may reduce the risk of recurrences compared to medical therapy [11,12]. The first randomized trials (RESPECT, CLOSURE I, PC TRIAL) failed to show significant superiority of PFO closure [13,14,15] over medical treatment. Antiplatelet therapy remained the default therapeutic strategy in PFO patients with cryptogenic stroke, even though the meta-analysis of these trials showed a statistically significant risk reduction in stroke in the PFO closure group (pooled HR = 0.59, 95%CI (0.36–0.97), *p* = 0.04) [16].

## 3. Change of Paradigm: Current Validated Indications for PFO Closure

Back in 2017, three positive randomized control trials (REDUCE, CLOSE and DEFENSE-PFO [17,18,19]) showed a significant clinical benefit, with a reduction in recurrent events after PFO closure compared to conservative therapy. The three-arm CLOSE study was the only one to randomize patients to either PFO closure or oral anticoagulation, and also showed a significant benefit to PFO closure [18]. One crucial difference between the CLOSE study and the previous trials was a better selection of higher-risk patients. Indeed, there were fewer specific inclusion criteria in the negative trials, leading to the potential inclusion of cerebral infarcts that were unlikely to be due to a PFO. Positive trials included younger patients (≤60 years) and introduced the notion of “high-risk PFO,” defined by the presence of ASA (>10 mm) or a pronounced right-to-left shunt in addition to the PFO. Consistent conclusions were finally reported in the results of the long-term RESPECT trial that started several years earlier [20]. 

**Figure 1:**
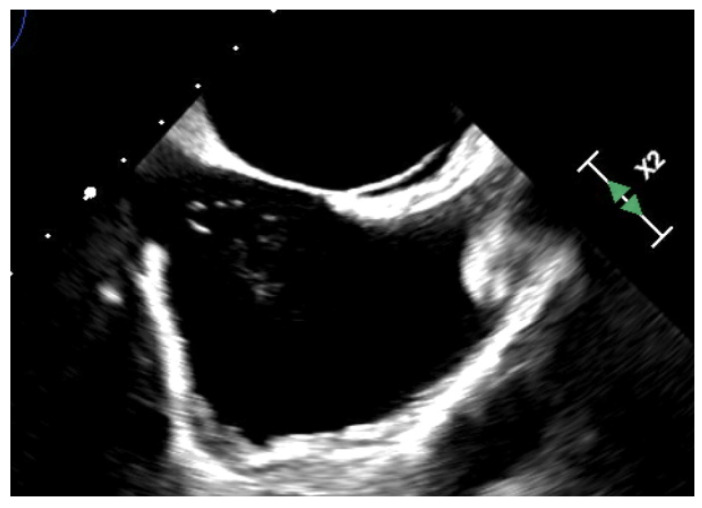
Chiari network.

Given those observations, European societies [21] suggest percutaneous closure of a PFO in selected patients aged 18 to 65 years with a confirmed cryptogenic stroke, TIA (transient ischemic attack) or systemic embolism; and an estimated high probability of a causal role of the PFO as assessed by clinical, anatomical and imaging features. It is thus essential to evaluate the probability of an embolism being PFO-related. Specific features should be sought:

- PFO is more often involved when patients are young without risk factors or causes of stroke [22,23].
- Cortical infarcts are more commonly embolic in origin compared to white matter infarcts.
- Causal role of PFO can be suspected in presence of ASA, shunt severity and an atrial septal hypermobility [17,19,20] as well as in PFO sizes, presence of Chiari network or Eustachian valve (Figure 1) [24,25,26,27].
- Simultaneous pulmonary embolism and/or DVT strongly suggests an implication of PFO [28,29,30].
- DVT, immobilization, long journeys, straining pre-stroke or obstructive sleep apnea can be associated with an implication of PFO [31,32].
- The RoPE score (Risk of of Paradoxical Embolism) has been validated and can be part of a comprehensive individual evaluation. The higher the score is, the greater the probability of the PFO being responsible for the stroke [33,34].

An updated nomenclature related to stroke risk and PFO has been proposed by the PFO International Workup Group, introducing the term “PFO-associated stroke” [35]. This entity means a superficial, large, deep or retinal infarct in the presence of a medium- to high-risk PFO without another identified cause. Cases are classified according to the probability of the PFO being the causative mechanism. The probability (highly probable, probable, possible or unlikely) is estimated based on several factors, such as visualization of a straddling thrombus, ASA, presence of large shunt, presence of pulmonary embolism or DVT.

Based on this definition, the newly published Society of Cardiovascular Angiography Intervention (SCAI) guidelines recommend PFO closure in patients aged between 18 and 60 with a history of PFO-associated stroke, but also in patients 60 years or older [36]. Systemic embolism in which other embolic etiologies have been excluded is a criterion for PFO closure. However, regarding a history of transient ischemic attack (TIA) without a prior PFO-associated stroke, SCAI guidelines differ from European guidelines and suggest against PFO closure. 

One challenging issue remains to properly rule out atrial fibrillation (AF). Indeed, recurrences of left circulation embolism may also be due to left atrial appendage thrombosis rather than to paradoxical embolism. AF frequently occurs without specific symptoms among patients with cryptogenic stroke, and can be difficult to detect [37]. In high-risk patients, use of insertable cardiac monitors (ICM) [38,39] or external ECG monitoring [40,41] can increase AF detection in cryptogenic stroke. Therefore, ICM can be considered to rule out AF before deciding on PFO closure in high-risk patients for AF [42].

## 4. Other Pathogenic Conditions Associated with PFO

### 4.1. Migraine

Prior history of migraine is frequent in PFO-associated stroke patients. Observational studies [12,43,44,45,46,47,48,49,50,51,52,53,54,55,56,57] show a significant improvement in migraine after PFO closure (Table 1), as shown in a recent meta-analysis (OR 0.18 [0.06–0.50]) [58], whereas individual randomized clinical trials (RCTs) did not show any statistically significant difference in their primary outcomes (i.e., responder rates or complete migraine resolution) (Table 2). On the contrary, a meta-analysis of secondary endpoints of these RCTs demonstrated a statistically significant reduction in migraine attack frequency and duration [59]. Particularly, subgroups of patients suffering from migraine with aura were statistically more improved with PFO closure compared to patients who received medical therapy (OR 0.21 [0.12–0.37]) [58]. Routine PFO closure for migraine is not currently indicated but can still be considered for clinical trials or in migraine with aura for compassionate use.

### 4.2. Arterial Hypoxemia and Platypnea Orthodeoxia Syndrome

A shunt through the PFO, mixing arterial and venous blood, can decrease oxygen in blood and cause arterial hypoxemia (SaO_2_ or SpO_2_ < 90% or PaO_2_ < 60 mmHg) with or without cyanosis. In most cases, the PFO shunt only aggravates pre-existing causes of hypoxemia. Diseases associated with elevated right heart pressure, such as pulmonary hypertension, obstructive sleep apnea syndrome (OSAS), chronic obstructive pulmonary disease (COPD), exercise desaturation or high-altitude pulmonary oedema (HAPO), can increase minor shunts. Positive pressure in mechanical ventilation may also increase the prevalence of PFO opening [66]. Platypnoea-orthodeoxia syndrome (POS) is characterized by dyspnea and arterial deoxygenation; it is typically induced by taking an upright position and relieved by lying supine [67]. In all syndromes, a lower-than-expected increase in or absence of SaO_2_ or SpO_2_ with FiO_2_ 100% suggests a significant intracardiac shunt. Due to their rarity, to date, there are no randomized trials assessing percutaneous closure of PFO in patients with desaturation syndromes.

A few small cases series studies have demonstrated stable relief of POS symptoms for up to five years post-closure, with improved arterial oxygen saturation in all patients without severe pulmonary hypertension [68,69,70]. Those results were confirmed in two larger registries with a 6% incidence of procedure-related complications [71,72]. Thus, in patients with PFO-related POS percutaneous closure of the PFO is recommended by European societies as a first-line treatment, in the absence of severe pulmonary hypertension [58].

In exertional desaturation, PFO closing seem to improve SaO_2_ or SpO_2_ in some observational studies [73,74]. Moreover, in a case-control study of 40 patients suffering from OSAS, PFO closure allowed statistically significant improvement in left ventricular diastolic function, in indices of apnea and desaturation episodes as well as a reduction in systemic arterial pressure [75]. These data suggest that percutaneous closure of PFO can potentially improve arterial oxygen saturation and symptoms in selected patients. 

Given the lack of strong evidence, PFO closure can be proposed after shared decision making, with clear evidence of the role of the PFO in desaturation.

### 4.3. Decompression Sickness (DCS)

Decompression sickness (DCS) is a complex condition caused by exposure to a hypobaric environment in situations such as flying at <350 mmHg barometric pressure or >18,000 ft altitude (altitude DCS) or returning to sea level after an ascent from depth (mining or diving).

A retrospective case-control study, with grouped analysis of recreational, military and professional divers, demonstrated the association between risk of DCS and PFO with an OR of 2.5 [76]. In other studies where recreational divers performed provocative diving that required decompression stops, DCS was reported to be five- to six-fold higher in divers with PFO [77,78]. In a meta-analysis of four studies comparing the prevalence of PFO in patients with DCS to that in patients without DCS, the presence of PFO with a right-to-left shunt was associated with risk of DCS with an OR of 5.63 (95% CI: 3.14–10.09).

In a recent cohort where PFO closure was offered to divers with high-risk PFO, there were no DCS events reported after closure; whereas, for those advised to dive conservatively, the number of DCS events remained higher (HR: 26.170; 95% CI: 5.797–118.16; *p* < 0.0001). However, in patients with low-shunt PFO who were advised to dive conservatively, the number of DCS events was similar to that in control patients without PFO [79]. 

Current positions of European and International societies on DCS and PFO are listed in Table 3.

### 4.4. Neurosurgery in Sitting Position

During neurosurgery, particularly in a sitting position, patients with PFO can experience paradoxical air embolism in up to 14% of cases. TOE, transcranial doppler or end tidal CO_2_ monitoring can be used to detect clinically significant venous air emboli [81,82]. However, such monitoring cannot prevent air embolism, and few studies have proposed preoperative PFO closure [83,84]. However, there are no published clinical studies, and timing of surgery post-intervention or duration of antiplatelet therapy remains unanswered. Given those observations, it is better to consider surgery in prone position when possible.

A summary of position statements for the controversial indications is presented in Table 4.

## 5. Procedure

PFO closure is performed in expert centers. The femoral vein is the preferred needle-puncture site for the procedure. Vena cava filter is not a contraindication for femoral access [85]; in rare cases of inferior vena cava (IVC) occlusion, jugular access is preferred.

Per procedural inter-atrial septum (IAS) imaging is essential to precisely assessing its anatomy, which can be complex, to improve the success rate. Indeed, imaging allows anatomic measurements in order to select the device’s size appropriately. Currently-used PFO closure devices are detailed in Table 5.

Width and length of the PFO tunnel, existence of an ASA, associated atrial septal defect (ASD), presence of Chiari network or Eustachian valve will help determine the best technical approach [86]. Indeed, challenging septal anatomies such as long tunnel (>12 mm) or CIA-like anatomy, enlarged aortic root, hypertrophic secundum septum or multiperforated septal aneurism are associated with more per-procedural complications, incomplete closure and higher risk of a recurrent event [87,88]. In some cases, a trans-septal puncture, larger or multiple devices may be used to obtain complete sealing [89,90]. Another interest of per-procedural imaging is to eliminate the presence of a thrombus inside the PFO, which should contraindicate the procedure. Aortic root visualization is also important to prevent pericardial effusion or aortic threatening. General anesthesia with transesophageal echocardiographic (TEE) guidance usually provides optimal image quality [91].

When local anesthesia is preferred, imaging is still required for optimal safety and optimal closure of the PFO, especially in complex anatomy. Intra-cardiac echocardiography (ICE) (which needs a second femoral puncture for the probe) [92,93] or micro-transesophageal echography can be used as a less invasive option [94]. 

Periprocedural anticoagulation is paramount to prevent thrombus formation on wires, sheaths, catheters, devices, or in the atria. Anticoagulation is usually achieved by the administration of intravenous (IV) unfractionated heparin (UFH) with a target activated clotting time of >250 s [95,96]. Use of enoxaparin 0,5 mg/kg is also safe and easy to use for structural heart intervention [97].

### Complications

Procedural complications of percutaneous closure include post-procedural atrial fibrillation, cardiac tamponade, aortic root injury or erosion, hemothorax, pneumothorax and vascular complications. Device complications include device thrombosis, device migration and nickel allergy. Table 6 represents the incidence of procedural complications.

A real-world retrospective study suggested that the rate of any adverse event was 10.9% in patients over the age of 60 and 4.9% in patients younger than 60 [98]. However, in the meta-analysis of the six RCTs comparing PFO closure and medical therapy, fewer complications were reported (2.40% (95% CI, 1.03–4.25; I2 = 77%)) and there was no difference in terms of mortality (13/1844 vs. 15/1667 deaths RR = 0.79, IC 95%: 0.39–1.60, *p* = 0.51) [99]. 

Vascular complications occurred in up to 30% of procedures, but these were usually mild, and only 3% needed surgical repair in one case series [100]. Cardiac tamponade, pneumothorax and hemothorax are very rare (<0.5%) [98]. 

**Table 6:** Incidence of procedural complications.

Complications	Type
Frequent (up to 20–30%) [101]	Minor vascular complications Atrial fibrillation
Rare (<1% [96,102].	PFO device embolization Device thrombosis
Very rare (<0.5%) [98].	Cardiac tamponade Pneumothorax Hemothorax

Post-procedural atrial fibrillation is a complication associated with an increased risk of recurrent events [103]. The underlying risk factors are not fully elucidated; they include the complicated nature of the procedure itself, local inflammation of the atrium, reentry circuits created by electrical obstruction due to the device or left atrial dysfunction that could have existed before the stroke. The mean incidence of AF after PFO closure was 3.2% in this meta-analysis of RCTs versus 0.47% in the medical arm [104]. In a retrospective cohort study with 1349 patients who underwent PFO closure, 3.9% patients developed new atrial flutter or AF [105] with most cases detected in the first four weeks following the procedure.

Nevertheless, the incidence of atrial fibrillation is probably underestimated, as shown in a recent study using ICM and external ECG monitoring, where the incidence reached up to 20.9% in the month following the procedure [101]. The determinants of new onset of atrial fibrillation were older age, male sex and use of larger device.

Incidence of PFO device embolization is very low (approximately 0.7%) and seems to be fostered by the presence of hypermobile septum primum or thick septum secundum [102]. Device thrombosis is also rare. In this study [96], thrombus formation evaluated with systematic TEE was found in 15 of the 593 (2.5%) PFO patients. Nevertheless, most of the nine devices evaluated are no longer marketed. There was no reported case in CLOSE nor RESPECT, and only two cases identified in REDUCE trial (0.5%), with good evolution under anticoagulation [96]. Some rare cases of nickel allergies have been described as responsible for various symptoms, such as nonspecific chest pain, dyspnea, migraine or hyperleukocytosis. Symptoms usually disappear with the device’s endothelialization, but can sometimes require steroid treatment or, rarely, the device’s extraction [106]. The Gore Helex Septal Occluder device seems to be less allergenic and can be used in these situations [107]. Unfortunately, there is no evidence yet on how to predict the risk of allergy in the case of a positive prick test.

## 6. Ongoing Challenges

### 6.1. Antithrombotic Treatment

Antiplatelet treatment regimen before PFO closure procedure is not yet standardized. There is no study evaluating the benefit of a loading dose of antiplatelet agent before percutaneous closure. In the CLOSE and RESPECT trials, all patients were treated on the day of the procedure with a double antiplatelet agent. Only patients in the REDUCE trial received a loading dose of CLOPIDOGREL 300 mg.

Post-procedure treatment is also poorly standardized. Consistent with the position of European society, ref. [108] decisions regarding post-procedural therapy should be made according to the strategies used in RCTs, which are summarized in Table 7.

There is no recommendation on antiplatelet duration beyond one month of dual antiplatelet therapy after PFO closure [36], due to the knowledge gap. The rational for the use of transient dual antiplatelet therapy following PFO closure is the prevention of device thrombosis and embolic event recurrence. However, in a recent international observational study including 1532 patients from seven centers in France and Canada [109], the use of dual antiplatelet strategy did not seem to change clinical outcomes compared to the use of single antiplatelet therapy.

### 6.2. Residual Shunts

The main results of studies evaluating residual shunts (RS) are presented in Table 8. In clinical practice, RS may be present in up to 25% of patients six months after PFO closure [110,111,112,113,114,115,116,117,118,119,120,121,122,123,124,125,126,126,127,128,129]. Use of larger devices (35 mm) seem to be associated with higher risk of residual shunts, as well as presence of an ASA, a multiperforated septum and short or very long tunnel. Impact on neurological recurrences remains unclear as studies on this specific issue have reported contrasting results.

### 6.3. Post-Procedural Atrial Fibrillation 

Management of post-procedural atrial fibrillation remains challenging and there is no specific recommendation on this topic. To assess the clinical consequences and the management of these arrhythmia events, the ongoing AFLOAT study (Assessment of Flecainide to Lower the patent foramen ovale closure risk of atrial fibrillation or Tachycardia, NCT05213104) will evaluate the potential benefit of flecainide to prevent arrhythmias after PFO percutaneous closure.

### 6.4. Patients > 60 Years-Old

Only the DEFENSE-PFO trial included few patients older than 60 years (the mean age was 50 years), while those patients were excluded from the other RCTs [17]. However, in older patients with a history of cryptogenic stroke, prevalence of the PFO is about twice higher than in patients with stroke of a known cause [130]. Bayes’ theorem indicates that a PFO is present in approximately 25% of elderly patients who have suffered a cryptogenic stroke, and could be the cause in approximately half of the cases [22]. In this study including patients older than 60 years [131], PFO closure was safe, and incidence of recurrent ischemic events after 3 years of median follow-up was relatively low compared with historical cohorts of patients treated medically. These data suggest that the diagnostic workup for an ischemic stroke in patients older than 60 years with undetermined cause should probably include the evaluation of the presence of PFO. However, in older patients, a higher risk of recurrent cerebrovascular events was observed compared to younger patients, partly due to the higher rate of cardiovascular risk factors and established coronary artery disease in this older group of patients which also increases the likelihood of underdiagnosed AF. CLOSE-2 (NCT05387954) will include 792 participants aged 60 to 80 years and will compare PFO closure plus antiplatelet therapy to antiplatelet therapy alone to prevent stroke recurrence. Inclusion criteria are PFO with large shunt (>20 microbubbles) or a PFO associated with an ASA (>10 mm), and an otherwise unexplained ischemic stroke. The trial will also evaluate whether oral anticoagulant therapy is superior to antiplatelet therapy. 

### 6.5. Delay from Last Ischemic Event

Randomized controlled trials evaluating PFO percutaneous closure included patients with a recent embolic event only: within 9 months prior randomization in the RESPECT trial, and within 6 months in the CLOSURE I, REDUCE, CLOSE and DEFENSE-PFO trials. In an international cohort [132], the 2-year rate of stroke or TIA in patients undergoing late PFO closure (≥6 months) was comparable to the event rate obtained in the early (i.e., <6 months) PFO group, and appears to be relatively low compared to theoretical 2-year recurrence rate as estimated by the RoPE score. This analysis provides indirect evidence that the delay from the last ischemic event does not impact outcomes following PFO percutaneous closure for secondary prevention.

## 7. Conclusions

PFO is implicated in the pathogenesis of several conditions. PFO closure is today the mainstay therapy for patients with cryptogenic stroke and concomitant high-risk PFO. This recommendation is based on results obtained in several RCTs and subsequent meta-analysis, in which the rate of recurrent stroke was much lower among PFO closure patients compared to those under antithrombotic treatment alone. Nevertheless, in other pathogenic conditions associated to PFO (migraines, decompression sickness, platypnea–orthodeoxia syndromes) the heterogeneity of patients is high, while evidence in favor of percutaneous closure is weak; therefore, individualized decisions remain often empiric. Further observational and randomized studies are warranted to clarify all the remaining issues in PFO closure. 

## Figures and Tables

**Table 1:** Observational studies evaluating migraine improvement in PFO closure.

Study	Prevalence of Migraine in Patients Referred for PFO Closure	Endpoint	% Migraine Improvement or Resolution	Follow-Up (Month)
Anzola et al., 2006 [60]	50/163 (31%)	Improvement of migraine score	88	12
Azarbal et al., 2005 [45]	37/89 (42%)	No. of patients with migraine	76	18
Biasco et al., 2014 [46]	217/835 (26%)	No. of patients with migraine	83	45
Donti et al.,2006 [47]	35/131 (27%)	No. of patients with migraine	91	20
Dubiel et al., 2007 [48]	46/191 (24%)	No. of patients with migraine	87	38
Giardini et al., 2006 [49]	35/131 (27%)	No. of patients with migraine	91	20.4
Jesurum et al., 2008 [50]	NA	No. of patients with migraine	77	9
Khessali et al., 2012 [51]	204/590 (35%)	No. of patients with migraine	76	12
Kimmelstiel et al., 2007 [52]	24/41 (59%)	Patients with migraine and migraine score	83	3
Morandi et al., 2003 [53]	17/62 (27%)	No. of patients with migraine	88	6
Papa et al.,2009 [54]	28/76 (37%)	No. of patients with migraine	82	12
Post et al.,2004 [61]	26/66 (39%)	No. of patients with migraine	65	6
Reisman et al., 2005 [55]	57/162 (35%)	No. of patients with migraine	70	12
Schwerzmann et al., 2004 [57]	48/215 (22%)	No. of migraine attacks	81	12
Vigna et al., 2009 [56]	NA	No. of migraine attacks	89	16
Wahl et al., 2010 [62]	150/603 (25%)	No. of migraine attacks	85	58
Wilmshurst et al., 2000 [43]	21/37 (57%)	No. of patients with migraine	86	30

**Table 2:** RCTs assessing PFO closure in migraine.

Trial	Number of Patients	Device	Follow-Up (Month)	Primary Endpoint	Results
MIST, Dowson et al., Circulation, 2008 [63]	432	Starflex	6	Cessation of migraine	No difference between implant and sham groups (3 of 74 versus 3 of 73) (*p* = 0.51)
PREMIUM, Tobis et al., JACC, 2017 [64]	230	Amplatzer PFO occlutech	12	50% reduction in migraine attacks	No difference in responder rate in the PFO closure versus control (45 of 117 vs. 33 of 103) (*p* = 0.32)
PRIMA, Mattle et al., EHJ, 2016 [65]	107	Amplatzer PFO occlutech	12	Reduction in monthly migraine days during months 9–12 after randomization	−2.9 days after PFO closure vs. −1.7 days in control group (*p* = 0.17)

**Table 3:** Current positions of European and International societies on DCS and PFO.

International Position Paper of Underwater Medicine Societies [80]	European Position Paper [58]
It is not recommended to systematically screen divers for the presence of PFO. In case of cerebral, spinal, vestibular or cutaneous episode of DCS, suspect PFO. When PFO is found, interpret results considering size, degree of patency (spontaneous or only after provocation maneuver) and the clinical/diving context of DCS. Causal relation between DCS and PFO is difficult to establish. Evaluate PFO treatment options: o Diving cessation o More conservative diving o PFO closure Careful consideration of the risks and benefits is needed when considering these options. Authorize resumption of diving after PFO closure only if: o Repeated contrast echography > 3 months after procedure confirms closure o Antiplatelet medication (except for aspirin) is stopped	Consider PFO closure in patients with history of DCS: If probability of causal PFO is high If flying/diving cessation is unthinkable (a) If production of venous gas emboli achieved by behavioral change is not possible (b) If the risk of recurrent DCS, despite conservative limits, is deemed unacceptable by the patient after consultation with an experienced dive or aerospace physician After PFO closure, document complete closure before authorizing unrestricted diving.

**Table 4:** Summary of position statements for the controversial indications.

Indication	Position Statements
Migraine	PFO closure for migraine can be considered for clinical trials or in migraine with aura for compassionate use
Desaturation syndromes	Propose PFO closure with shared decision making, if no other factor involved in the desaturation syndrome
Decompression sickness	Consider PFO closure in patients with history of DCS when flying/diving cessation is not an option
Neurosurgery in sitting position	Consider surgery in prone position

**Table 5:** PFO closure devices.

Device	Size (Right/Left Atrial Disc)	Sheath Size
AMPLATZER PFO OCCLUDER (Abbott)	18 mm (18/18) 25 mm (25/18) 30 mm (30/30) 35 mm (35/25)	8F 8F 8F 9F
AMPLATZER MULTIFENESTRATED SEPTAL OCCLUDER-Cribriform (Abbott)	18 mm (18/18) 25 mm (25/25) 30 mm (30/30) 35 mm (35/35) 40 mm (40/40)	8F 8F 8F 9F 9F
FIGULLA FLEX II (Occlutech)	18 mm (18/16) 25 mm (25/23) 30 mm (30/25) 35 mm (35/31)	7F 9F 9F 11F
CARDIOFORM SEPTAL OCCLUDER (Gore)	20 mm (20/20) 25 mm (25/25) 30 mm (30/30)	10F 10F 10F

**Table 7:** Antiplatelet therapy in RCTs.

Trial	Antiplatelet Therapy
CLOSURE-1	Clopidogrel (75 mg) for 6 months + Aspirin (81–325 mg) for 2 years
PC TRIAL	Aspirin (100–325 mg) for 5 to 6 months + Ticlopidine (250–500 mg) or Clopidogrel (75–100 mg) for 1 to 6 months
RESPECT	Clopidogrel for 1 month + Aspirin for 6 months, followed by mono-antiplatelet therapy upon investigator’s choice
REDUCE	Clopidogrel 300 mg before or after the procedure, followed by Clopidogrel 75 mg for 3 days, and antiplatelet therapy until the end of the study
CLOSE	Clopidogrel + Aspirin for 3 months, followed by antiplatelet therapy until the end of the study
DEFENSE-PFO	Clopidogrel + Aspirin for at least 6 months, followed by antiplatelet therapy or anticoagulation upon investigator’s choice

**Table 8:** Studies evaluating RS.

Study	Prevalence of RS at 6 Month Follow-Up	Risk Factors for RS	Impact on Neurological Recurrences
Trabattoni, 2023 [110]	3/442 (0.6%) *	NA	2 patients had recurrent TIA (without RS)
Deng, 2020 [111]	243/1078 (22.5%) ^$^	NA	RS was associated with an increased risk for stroke or TIA recurrence
lu He, 2020 [112]	3/268 (1.1%)	Persistent RS during normal breathing and the Valsalva maneuver	2 patients had recurrent TIA (1 with trace RS and 1 without RS)
Alexia Karagianni, 2020 [113]	65/282 (23.7%)	NA	The risk ratio of rCVEs in patients with RS was 2.9 times higher than it was in patients without RS (95% CI: 1.4–6.1) at follow-up visit
Eyal Ben-Assa, 2020 [114]	29/110 (26.3%)	NA	NA
Wintzer-Wehekind, 2019 [115]	6/183 (3.3%)	NA	2 stroke events (1 related to atherothrombotic disease and 1 due to a vertebral artery dissection)
Roel J R Snijder, 2019 [116]	60/250 (24%) ^$^	NA	Within the first 12 months, recurrent events occurred in 5 patients (2 with RS)
Moon, 2019 [117]	10/38 (26%) *	NA	NA
Cheli, 2015 [118]	20/120 (17%)	Post-procedural shunt, use of a bigger device (35 vs. 25 mm)	Neurological recurrences (1 stroke, 6 TIA) were equally distributed between the groups
Biasco, 2014 [46]	16/70 (22.8%)	NA	NA
Matsumura, 2014 [119]	17/167 (10.2%) ^$^	NA	NA
Caputi, 2013 [120]	78/401 (19.5%)	NA	33.3% of patients with neurological events had RS
Butera, 2013 [121]	27/525 (5.1%) ^$^*	NA	NA
Marchese, 2013 [122]	17/127 (13.3%) ^$^	Presence of ASA and longitudinal FO dimension > 20.8 mm	NA
Wallenborn, 2013 [123]	146/1775 (8.2%) ^#^	NA	54/63 events occurred in patients without RS. HR 1.7 [0.8–3.6], *p* 0.16
Jochen Wöhrle, 2012 [124]	78/267 (29%)	NA	There was no stroke or TIA during follow-up
Sherman G Sorensen, 2012 [125]	21/135(7%) *	width of the left atrial opening and balloon size	NA
Christoph Hammerstingl, 2011 [126]	22/127 (17.7%)	Helex occluder, PFo- canal- length and extend of atrial-septal-aneurysm	All ischemic events occurred in patients without incidence of RS
Tulio Diaz, 2010 [127]	21/424 (5%) *	24-hr postprocedure shunt	No deaths, strokes or TIAs were reported in patients with RS
Matthias Greutmann, 2009 [128]	26/135 (17%)	atrial septal aneurysm, devices’s size patients treated (35 mm vs. 25 mm)	2 patients (1.5%) had recurrent ischemic events during follow-up (1 had RS)
Nabil A Shafi, 2009 [129]	10/51 (19.6%)	PFO canal lengh	No short-term increased risk of TE events

* only moderate or severe residual shunting was adjugated; ^$^ at 1 year follow-up; ^#^ date of follow-up unknown; rCVEs: recurrent cardiovascular event; TE: thromboembolic.

## Data Availability

Not applicable.
